# Do Seminal Isoprostanes Have a Role in Assisted Reproduction Outcome?

**DOI:** 10.3390/life11070675

**Published:** 2021-07-10

**Authors:** Giulia Collodel, Daria Noto, Cinzia Signorini, Laura Gambera, Anita Stendardi, Amra Mahmutbegovic, Lucia Micheli, Andrea Menchiari, Elena Moretti

**Affiliations:** 1 Department of Molecular and Developmental Medicine, University of Siena, 53100 Siena, Italy; giulia.collodel@unisi.it (G.C.); cinzia.signorini@unisi.it (C.S.); amra.mahmutbegovic@student.unisi.it (A.M.); elena.moretti@unisi.it (E.M.); 2 Fertility Center, AGI Medica, 53100 Siena, Italy; lauragambera@agimedica.it (L.G.); anitastendardi@agimedica.it (A.S.); 3 Department of Medicine, Surgery and Neurosciences, University of Siena, 53100 Siena, Italy; lucia.micheli@unisi.it; 4 Department of Business and Law, University of Siena, 53100 Siena, Italy; andrea.menchiari@unisi.it

**Keywords:** embryo quality, in vitro fertilization outcome, male infertility, seminal F_2_-isoprostanes, sperm chromatin

## Abstract

F_2_-isoprostanes (F_2_-IsoPs), stereoisomers of prostaglandin F2α generated by the free radical-induced oxidation of arachidonic acid, have been associated with different male infertility conditions. This study aimed to evaluate the role of seminal isoprostane levels and sperm characteristics in the reproductive outcome and embryo quality of 49 infertile couples. Semen analysis was performed following WHO guidelines. Sperm chromatin maturity was detected using an aniline blue (AB) assay, and DNA integrity was assessed using the acridine orange (AO) test. Seminal F_2_-IsoP levels were quantified by gas chromatography/negative ion chemical ionization tandem mass spectrometry (GC/NICI–MS/MS) analysis. Correlations among variables and their impact on in vitro fertilization (IVF) and intracytoplasmic sperm injection (ICSI) outcome were investigated. F_2_-IsoP levels are positively correlated with double-stranded DNA sperm (*p* < 0.001) and negatively correlated with mature sperm chromatin (*p* < 0.001). Patients with positive outcomes had an increased percentage of sperm with double-stranded DNA, as did patients producing high-quality embryo, who showed higher F_2_-IsoP levels compared to those detected in the low-quality embryo group. An intriguing relationship between a mild increase in F_2_-IsoP levels, DNA integrity, and embryo quality seems to indicate that the non-enzymatic oxidation of arachidonic acid can be also a marker of metabolic activity in human semen.

## 1. Introduction

The life and death of mammalian spermatozoa are mostly ruled by oxidative stress (OS). It is well known that spermatozoa are capable producers of reactive oxygen species (ROS), derived from three potential sources, as revised by Aitken [1]: sperm mitochondria, cytosolic L-amino acid oxidases, and plasma membrane nicotinamide adenine dinucleotide phosphate oxidases.

Small amounts of ROS such as the superoxide anion, hydrogen peroxide, and nitric oxide are important for physiological sperm function, including capacitation, hyperactivation, and acrosomal reaction [2].

However, excessive production of ROS can play a negative effect on sperm concentration, motility, and morphology. In addition, high ROS levels may damage sperm DNA, RNA transcripts, and telomeres and, therefore, might represent a common underlying etiology of male infertility and recurrent pregnancy loss [3]. Polyunsaturated fatty acids (PUFAs), particularly rich in sperm membranes, represent the main target of the free radical insult, leading to oxidative lipid deterioration, the disruption of membrane characteristics that are critical in maintaining sperm function, including egg fertilization [1,4].

Aldehydes, such as malondialdehyde and 4-hydroxynonenenal; conjugated diene compounds; and isoprostanoids (a class of prostanoid isomers) are the secondary products of lipid peroxidation (LPO), and they have been investigated as seminal markers of oxidative damage [5,6]. Altered levels of F_2_-isoprostanes (F_2_-IsoPs), isoprostanoids that are stereoisomers of prostaglandin F2α (PGF2α) generated by the free radical-induced oxidation of arachidonic acid, have been associated with different male infertility conditions [7,8,9]. Seminal isoprostanes are negatively correlated with sperm motility and morphology [10,11] and positively correlated with sperm immaturity [12,13].

Assisted reproductive technology (ART) is used to treat infertility and involves procedures such as in vitro fertilization (IVF) and intracytoplasmic sperm injection (ICSI). One of the key determinants for success in ART is the embryo quality. Several studies explored the effect of oocyte parameters on embryo quality, but more intriguing is the study of the role of sperm quality in achieving the best embryos [14]. The conventional parameters used to assess semen quality are used in this study to choose treatments for couples undergoing ART, but they are not reflected in the fertilization rates, emphasizing their limited utility in the assessment of sperm function [15]. Sperm chromatin structure and sperm DNA integrity represent further important characteristics for successful fertilization and embryo development. Abnormal spermatozoa contain high levels of damaged DNA and show altered replacement of histones with protamines and chromatin packaging. These anomalies, often concomitant with high ROS concentration and low antioxidant capacity, are reflected as poor semen quality and poor reproductive outcome in infertile men [16]. Moreover, an increased percentage of sperm with immature chromatin has been associated with a low fertilization rate and slow embryo development [17].

This paper aims to evaluate the role of seminal isoprostane levels and sperm characteristics, such as chromatin status and DNA integrity, in the reproductive outcome and embryo quality.

## 2. Materials and Methods

### 2.1. Patients

Semen samples were obtained from the male partners (age 29–37 years) of 49 infertile couples referred to the AGI Medica IVF Center (Viale Toselli, 94/F, 53,100 Siena). The inclusion criteria for this study were idiopathic male factor infertility, and one year or more of unprotected intercourse without conception. The selected patients showed normal karyotypes and hormonal levels, the absence of systematic sperm defects, negative semen bacteriological cultures, and no varicocele diagnosis or leukocytospermia.

The female partners (age 27–35 years) had normal karyotypes and did not show ovulatory, tubal, and hormonal pathologies.

Before the sperm selection required for IVF or ICSI, 0.5–1 mL of seminal fluid was collected for sperm analysis. After semen analysis, the samples were centrifuged (15 min 400× *g*) to separate the spermatozoa from the plasma, and the latter was frozen at −80 °C for the evaluation of seminal F_2_-IsoP levels. Before freezing, butylhydroxytoluene (BHT) was added as an antioxidant (final concentration 90 μM).

The participants signed informed written consent before participation in this research, declaring acceptance that their semen samples and the clinical data might be used for scientific purposes.

### 2.2. Semen Analysis

The participants abstained from intercourse and masturbation for 3–5 days before the sperm collection. The semen samples were collected by masturbation into a sterile container, and the samples were examined after liquefaction for 30 min at 37 °C. Volume, pH, concentration, morphology, and motility were assessed as recommended by World Health Organization guidelines [18]. The sperm morphology was evaluated using the Papanicolaou test modified for spermatozoa [18].

### 2.3. Sperm Preparation

In cases of spermatozoa showing almost total absence of progressive motility, the semen was simply washed in Flushing Medium (Origio, Måløv, Denmark) before being used for ICSI.

In other patients, the standard swim-up technique was used to collect motile and active spermatozoa for IVF and ICSI. The sperm samples were washed with 2 mL of a Flushing Medium (Origio, Denmark) and centrifuged at 200× *g* for 10 min. The supernatant was discarded, and the pellet was gently over-layered with 0.5–0.8 μL of the medium; the tubes were inclined at 45° and kept at 37 °C and 5% CO_2_ for 45–60 min. Then, 0.2 μL of the uppermost medium containing motile sperm was aspirated with a sterile pipette. Concentration, motility, and morphology were recorded, and the sample was kept at 37 °C until use.

### 2.4. Ovarian Stimulation

The women were treated with an antagonist protocol. According to the patients’ age and body weight, the initial dose of gonadotropin was determined to be 150–400 IU/day.

Monitoring started on day 5 of the stimulation, and the dose of gonadotropin was adjusted according to the estradiol (E2) serum concentration and ovarian response, which were examined by ultrasound. When the leading follicles reached 13 mm in diameter, 0.25 mg of Cetrorelix (Merck-Serono, Darmstadt, Germany) was subcutaneously added daily until the day of hCG administration. An intramuscular injection of hCG was made when a minimum of three follicles reached a diameter of ≥18 mm. Oocyte pickup was performed by the ultrasound guide 36 h after the hCG injection.

### 2.5. Acridine Orange (AO) Assay to Evaluate Sperm DNA Susceptibility to Damage

Acridine orange (AO, 3, 6-bis (dimethylamino) acridine, hemi (zinc chloride) salt, BDH Chemicals Ltd., Poole, England) treatment assays are used to assess the sperm DNA vulnerability to acid-induced denaturation in situ by quantifying the metachromatic shift of AO fluorescence from green (double-stranded DNA) to red (denatured DNA). The AO test was carried out as described by Moretti et al. [19]. The slides were observed and scored with a Leitz Aristoplan fluorescence Microscope (Leica, Wetzlar, Germany) equipped with a 490 nm excitation light and 530 nm barrier filter. Three hundred sperm nuclei for each sample were analyzed and classified as green or red (sometimes, orange yellow) depending on fluorescence. The sperm heads that exhibited a green stain had double-stranded DNA; the sperm heads showing a spectrum of yellow orange to red fluorescence had single-stranded DNA. The results were expressed as the percentage of spermatozoa that showed normal DNA (green fluorescence).

### 2.6. Aniline Blue (AB) Test

This test verifies the maturity of the sperm chromatin, since AB shows binding affinity with lysine residues, an amino acid in which histones are rich. It is well known that, during the maturation process, spermatozoa replace most histones with protamines, basic proteins rich in arginine accomplishing chromatin compaction. Therefore, the blue staining of sperm after AB treatment indicates the persistence of histones and the immaturity of spermatozoa.

Two hundred microliters of seminal fluid were washed in phosphate buffer saline (PBS), centrifuged at 400× *g* for 15 min, and resuspended in PBS; the sample, appropriately diluted, was smeared on slides and air-dried. The slides were fixed with 3% glutaraldehyde in PBS, pH 7.2, for 30 min in a humid chamber at room temperature. Then, the slides were treated for 5 min with AB solution (5% aniline powder and 4% acetic acid, with solution made up to volume with distilled water; pH 3.5). After washing in distilled water, the slides were observed under a Leitz Aristoplan microscope (Leica, Wetzlar, Germany). Approximately 300 spermatozoa per sample were examined.

### 2.7. Determination of Total F_2_-IsoPs

Since F_2_-IsoPs are initially formed in situ on phospholipids (esterified F_2_-IsoPs) and later released into biological fluids as unesterified F_2_-IsoPs (free F_2_-IsoPs), the levels of total F_2_-IsoPs represents the sum of free and esterified F_2_-IsoPs.

At the time of total F_2_-IsoP assay, all seminal fluid samples were subjected to basic hydrolysis by incubation at 45 °C for 45 min in the presence of 1N KOH. Subsequently, all samples were acidified to pH 3 by the addition of 1N HCl, and tetradeuterated prostaglandin F2α (PGF2α-d4, 500 pg), used as an internal standard, was added to each sample.

After a period (15 min) of stabilization in the presence of the internal standard, the samples were purified by solid-phase extraction procedures that, by protocol, involve the three sequences of conditioning, washing, and elution. For seminal fluid samples, an octadecylsilane cartridge (C18, 500 mg, 55–105 μm Particle Size, 6cc, Waters, Milford, MA, USA) was used, through which, after an appropriate conditioning step, the samples were passed, followed by cartridge washes and elution.

Eluates from the C18 cartridge were transferred to a previously conditioned aminopropyl cartridge (NH_2_, 500 mg, 55–105 μm particle size, 6cc, Waters, Milford, MA, USA). After loading the eluate, washes and final elution were performed. The final eluates were collected and then subjected to a derivatization procedure after evaporating the solvent mixture under nitrogen blowing. Specifically, working on the dry residue of the extract, the carboxyl groups of F_2_-IsoP and PGF2α-d4 were derivatized to form pentafluorobenzyl esters, while the hydroxyl groups were converted to trimethylsilyl ethers.

Thereafter, total F_2_-IsoPs were determined in semen samples by gas chromatography/negative ion chemical ionization tandem mass spectrometry (GC/NICI–MS/MS). The derivatized samples were injected (2 μL) into the gas chromatograph (Trace GC and PolarisQ, Thermo/Finnigan, San Jose, CA, USA) to initiate the identification and quantification of F_2_-IsoP by GC/NICI–MS/MS. The ions determined in GC/NICI–MS/MS were the mass-to-charge ratio (*m*/*z*) 299 and m/z 303 product ions derived from the [M-181]^−^ precursor ions of F_2_-IsoPs (*m*/*z* 569) and PGF_2_α-d4 (*m*/*z* 573), respectively [20]. For calibration, commercial molecules were used (8-iso-PGF_2_α, also named 15-F_2_t-IsoP, Cayman Chemical, Item No. 16350, as reference molecule for F_2_-IsoPs; PGF_2_α-d4 Cayman Chemical, Item No. 316010, as internal standard).

### 2.8. Oocyte Preparation and ICSI and IVF Procedure

Oocytes were recovered from follicular fluid immediately after follicle aspiration. They were washed in Flushing Medium (Origio, Denmark) and then in Sage 1-Step TM with HSA (Origio, Denmark) and were finally incubated in 30 μL micro-drops of the same medium under Mineral Oil (Origio, Denmark) at 37 °C and 6% CO_2_. In four to five hours, cumulus cells were removed using hyaluronidase 20 IU (Origio, Denmark) and microinjected as described by Palermo et al. [21] using a Narishige micromanipulator (IM9B, Narishige, Tokyo, Japan). Eighteen hours after ICSI, the presence of two pronuclei and two polar bodies was checked to verify normal fertilization. The embryos were cultured in Sage 1-Step TM with HSA (Origio, Måløv, Denmark) until embryo transfer, 3 days after oocyte retrieval.

In the case of conventional IVF, the oocytes with cumulus cells were recovered in a dish with the selected spermatozoa (250,000 sperm/mL).

### 2.9. Embryo Score

Before the transfer, the embryos were evaluated morphologically and graded according to a scale [22] from 1 (good-quality embryo) to 4 (poor-quality embryo) based on the assessment of the presence/absence of cytoplasmic fragments, number, and symmetry of blastomeres. In this study, the embryos graded on Veeck’s scale as 3 and 4 were considered the group with low-quality embryos and those graded as 1 and 2 belonged to the group with good-quality embryos.

### 2.10. Statistical Analysis

The data were analyzed using SPSS v. 23.0 software package. A significance level of *p* ≤ 0.05 was adopted. Initially, the Kolmogorov–Smirnov test was used to verify the normal distribution of the variables. To assess the equality of variances or homoscedasticity, Levene’s test was performed. To compare the means between independent groups Student’s *t*-test was used.

Spearman’s correlation coefficient was employed to evaluate the correlation between the variables. For multiple comparison, the Bonferroni correction was adopted (*p* < 0.002).

Finally, a binary logistic regression stepwise analysis was conducted on a positive fertilization outcome and embryo quality to identify the variables predictors.

## 3. Results

The seminal F_2_-IsoP concentrations were measured in the semen samples used on the day of ART. In the same samples, semen volume, sperm concentration, progressive motility, and morphology (WHO, 2010) [18] were assessed; in addition, the sperm chromatin status (AB assay) and DNA integrity (AO test) were evaluated. The means and standard deviations of the variables considered in 49 infertile men are reported: volume 3.82 ± 1.55 mL, sperm concentration 47.51 ± 48.27 sperm/mL × 10^6^, sperm progressive motility 40.43 ± 18.22%, normal sperm morphology 8.16 ± 5.61%, double-stranded DNA 83.97 ± 15.99%, mature chromatin 68.69 ± 16.02%, and F_2_-IsoP 25.71 ± 10.81 ng/mL.

**Figure 1:**
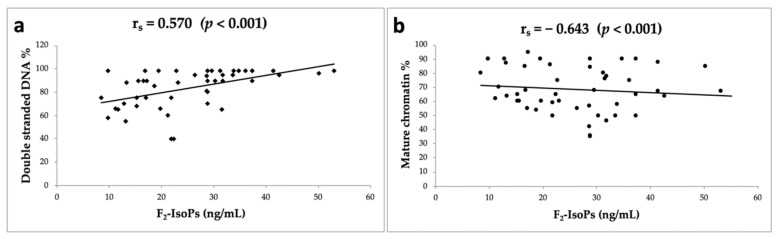
Correlations (rho Spearman’s coefficient) between F_2_-IsoPs (ng/mL) and double-stranded DNA % (**a**) and between F_2_-IsoPs (ng/mL) and mature chromatin (**b**) (in 49 infertile men) are shown.

All variables considered were correlated (rho Spearman’s coefficient), and the results are shown in Table 1. Interestingly, the F_2_-IsoP levels were positively correlated with double-stranded DNA sperm (*p* = 0.000, Figure 1a) and negatively correlated with mature sperm chromatin (*p* = 0.000, Figure 1b). As expected, progressive motility was positively correlated with sperm concentration (*p* = 0.000) and normal morphology (*p* = 0.001). The percentage of normal sperm morphology was positively correlated with sperm concentration (*p* = 0.000) and the percentage of sperm with mature chromatin (*p* = 0.001).

**Figure 2:**
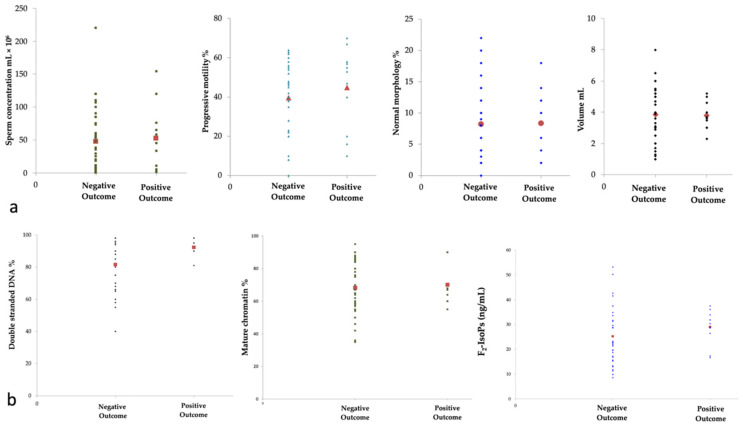
Scatter plots of the variables considered in 49 patients grouped according to assisted reproduction technique (ART) outcome: positive outcome (11 patients) and negative outcome (38 patients). (**a**) sperm concentration (mL × 10^6^), progressive motility %, normal morphology %, volume (mL) are shown; (**b**) double stranded DNA %, mature chromatin % and F_2_-IsoPs (ng/mL) are reported. The red symbols represent the mean of the values. The group with positive outcomes showed increased double stranded DNA % (**b**) than that observed in the group with negative outcomes (*p* = 0.002).

Then, the patients were grouped according to positive or negative outcome in ART (Figure 2a,b). The group with positive outcomes consisted of 11 patients (age 30–36 years), in which 7 of them had performed IVF and 4 had performed ICSI. The group with negative outcomes comprised 38 patients (age 29–37 years); among them, 23 had performed ICSI and 15 have performed IVF. The two groups showed similar F_2_-IsoP levels; however, the percentage of sperm with double-stranded DNA increased in patients with positive outcomes (*p* = 0.002, Figure 2b).

In both groups (data not shown), the F_2_-IsoP levels were negatively correlated with the percentage of sperm with mature chromatin and positively correlated with the percentage of sperm with double-stranded DNA, as in the whole patient population. To identify the predictors of a positive fertilization outcome, a binary logistic regression analysis was conducted, and the results are reported in Table 2.

A weak association between progressive motility (OR 1.11, 95% CI 1.01 to 1.22, *p* = 0.031) and the presence of double-stranded DNA% (OR 1.13, 95% CI 1.01 to 1.25, *p* = 0.026) with the outcome of assisted fertilization was observed.

**Figure 3:**
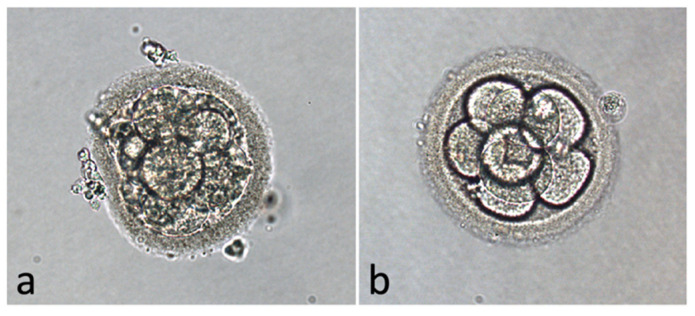
A poor-quality embryo (Veeck’s scale as 3 and 4) (**a**) and a good-quality embryo (Veeck’s scale as 1 and 2) (**b**) on day 3 after fertilization are shown. In (**a**), the embryo presents uneven-sized blastomeres, non-homogenous cytoplasm, and 35–50% fragments. In (**b**), the embryo presents even sized blastomeres.

Finally, we grouped patients according to embryo quality: the group with low-quality embryos (18 patients, Veeck’s scale [22] grades 3 and 4, Figure 3a) and the group with good-quality embryos (30 patients, Veeck’s scale [22] grades 1 and 2, Figure 3b). One couple did not obtain any embryo.

**Figure 4:**
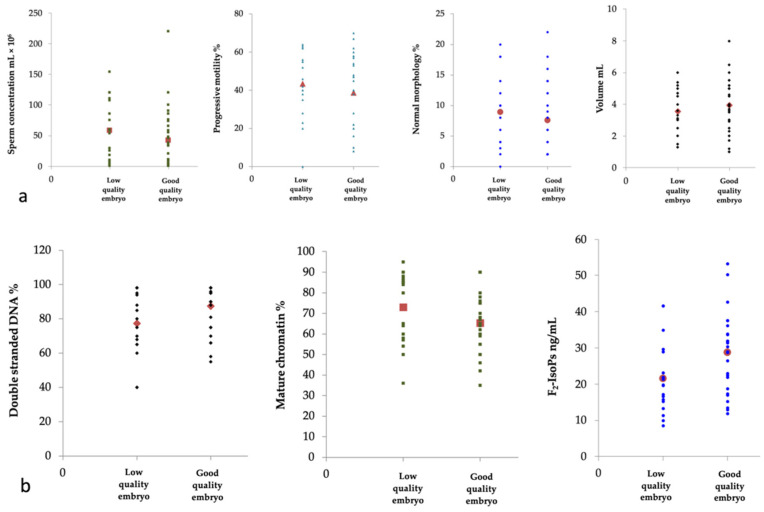
Scatter plots of the variables considered in 48 patients grouped according to embryo quality: low-quality embryos (18 patients) and good-quality embryos (30 patients). (**a**) sperm concentration (mL × 10^6^), progressive motility %, normal morphology %, volume (mL) are shown; (**b**) double stranded DNA %, mature chromatin % and F_2_-IsoPs (ng/mL) are reported. The red symbols represent the mean of the values. The group with good-quality embryos showed increased double stranded DNA % (**b**, *p* = 0.046) and F_2_-IsoP levels (**b**, *p* = 0.024) compared to those observed in the group with low-quality embryos.

The semen variables were compared, and the results are shown in Figure 4.

Semen samples producing good-quality embryos showed a higher percentage of sperm with double-stranded DNA (*p* = 0.046; Figure 4) and increased F_2_-IsoP levels (*p* = 0.024; Figure 4) compared to those obtaining low-quality embryos. In the group with good-quality embryos, a positive correlation (data not shown) was highlighted between the percentage of sperm with mature chromatin and semen parameters (sperm concentration, progressive motility, and normal morphology, all *p* = 0.000). In both groups, the F_2_-IsoP levels negatively correlated with the percentage of sperm with mature chromatin (group with low-quality embryos *p* = 0.008; group with good-quality embryos *p* = 0.000) and positively correlated with the percentage of sperm with double-stranded DNA% (group with low-quality embryos *p* = 0.032; group with good-quality embryos *p* = 0.000).

A weak association between semen F_2_-IsoP levels (OR 1.08, 95% CI 1.01 to 1.15, *p* = 0.019) and embryo quality was obtained (Table 3).

## 4. Discussion

The relevance of isoprostanes has been demonstrated in many pathological conditions [23], and their role in semen has received growing interest in the field of male infertility.

Recent surveys have reported that seminal 8-iso-PGF2α levels were significantly higher in infertile patients than in fertile men [10,11,12,24,25]. In particular, the levels of these compounds were particularly high in patients affected by varicocele, a pathology characterized by sperm immaturity [12,25].

In this research, we found that F_2_-IsoPs are a sensitive marker of sperm immaturity, detected with aniline blue assays, which evaluate the replacement of histones with protamines, confirming results previously obtained by transmission electron microscopy (TEM) analysis [12,13,25]. In brief, some ultrastructural characteristics of sperm immaturity are the presence of uncondensed chromatin, the presence of cytoplasmic residues, and coiled tails. A relationship between immaturity and increased F_2_-IsoPs levels was also reported in a case of globozoospermia [13], a genetic condition of male infertility characterized by sperm with round heads, the absence of acrosome, and immature chromatin. Defects in sperm chromatin, in particular alterations in histone/protamine replacement, has been associated with low-quality embryo affecting early embryo development [26], and increased sperm DNA fragmentation [27]. Overall, these results suggest that F_2_-IsoP levels are an efficient marker of defective histone replacement, suggesting that sperm maturation could be considered a process related to PUFA content and oxidative metabolism.

The seminal F_2_-IsoPs levels of the individuals considered in this research were similar to those detected in groups of patients affected by oligoasthenoteratozoospermia (23.42 ± 8.36) [10] and idiopathic infertility (24.30 ± 50.4) [12]. These levels were slightly higher than those detected in fertile men [8,9,12] and lower than those measured in semen samples from patients affected by varicocele [8,9,12].

The novelty of this study was the investigation of the relationship between semen isoprostanes, fertilization outcome, and embryo quality. The literature on this topic is very scant; to the best of our knowledge, only one research reported that the 8-iso-PGF_2α_ levels, evaluated in the urine of men from couples attending fertility treatment, did not show significant associations with reproductive outcomes [28].

The intriguing results of the present research regards the positive effect of F_2_-IsoPs on DNA susceptibility to the damage tested with an acridine orange assay.

In addition, comparing the variables in the two groups based on embryo quality, we observed that the group with good-quality embryos showed both increased percentages of sperm with double-stranded DNA and, unexpectedly, slightly higher F_2_-IsoP levels than the group with low-quality embryos (both *p* < 0.05).

Analyzing the odds ratio of the variables, a slight influence of F_2_-IsoP levels on embryo quality was confirmed; however, no significant influence was found for double-stranded DNA. This discordant result could be due to the characteristics of the samples considered in this study. DNA integrity seems to play a role in the positive outcome of fertilization. Numerous studies investigated the relationship between DNA integrity and ART outcome [29], even if the results are contradictory, considering different factors such as patient selection, differences in DNA integrity detection, female age, and others. Agarwal et al. [29] reviewed many studies on this topic and concluded that DNA integrity is detrimental to normal fertilization, embryo development, and the success of ART, suggesting that sperm DNA testing is important in male infertility evaluation.

The determination of F_2_-IsoPs represents one of the most reliable approaches to evaluating a condition of endogenous LPO in vivo, and F_2_-IsoPs are considered markers in many pathologies including reproductive ones in which OS may be involved, such as varicocele and leukocytospermia, suggesting that arachidonic acid oxidation/metabolism may influence different male reproductive pathological conditions. However, the results obtained are oriented in another direction that needs to be discussed. First, F_2_-IsoPs are not mere markers of OS, but they also elicit a wide variety of responses in different cell types [23,30]. Evidence of their biological activities are reported in the literature [31,32,33]. In addition, the F_2_-IsoP receptor, thromboxane A2 receptor [31], has been localized in rat spermatids, cells that undergo relevant chromatin modifications [34], and has been demonstrated to be involved in the regulation of gene expression in murine Leydig cells [35]. Then, a potential biological activity of F_2_-IsoPs in the male reproductive system cannot be excluded.

For these reasons, we can hypothesize that the sperm that obtained better embryo quality are those active from a metabolic point of view, so they produce a certain amount of ROS, leading to a moderate increase in F_2_-IsoPs detected in the semen. Therefore, the role of F_2_-IsoPs toward spermatozoa can be different compared to other cells and tissues; F_2_-IsoPs could trigger relevant physiological events as capacitation, and only high concentrations of these molecules play a detrimental effect. These observations agree with those of Pasqualotto et al. [36], who analyzed LPO and total antioxidant capacity (TAC) in follicular fluids. They found that LPO and TAC were lower in patients who did not become pregnant than in patients who did, concluding that LPO may represent a good marker of metabolic activity within the follicle and may be necessary to establish pregnancy.

In infertile patients without inflammatory pathologies, a mild increase in F_2_-IsoP levels could enhance the activation and regulation of the Cation channel of Sperm, CatSper [37,38,39,40], and could play a relevant physiological role in the fertilization outcome and embryo quality.

In addition, the sperm membrane of infertile men showed an altered lipid profile [41] with consistent amounts of arachidonic acid [25] that can negatively affect the capacitation process. A mild increase in isoprostanes in semen with respect to the data reported from fertile subjects [9] suggested that the presence of moderate OS, probably slightly higher than the physiological one, is able to increase LPO but unable to damage DNA. Moderate ROS levels can also be hypothesized by observing the values of progressive motility in our cases that, although decreased with respect to reference values [18], are not severely reduced.

High ROS concentrations are dangerous to spermatozoa, but these gametes evolved the capacity to generate highly reactive molecules as they need them to undergo capacitation and to activate motility [1]. Zhang et al. [42] demonstrated a close association between mitochondrial function, acrosin activity, and the DNA fragmentation index in human spermatozoa. This evidence seems to suggest that mitochondrial functional integrity is necessary to maintain sperm fertilizing ability, also influencing DNA integrity, which is a prerequisite for a high fertilization rate and embryo quality after IVF and ICSI [43,44,45].

## 5. Conclusions

The intriguing relationship between a mild increase in F_2_-IsoP levels, DNA integrity, and good embryo quality seems to indicate that F_2_-IsoPs can be a marker of metabolic activity in human semen. We are aware that these are preliminary observations that must be confirmed by other studies, but this possibility deserves attention as it may represent a new role of F_2_-IsoPs in human semen.

## Figures and Tables

**Table 1:** Correlations (rho Spearman’s coefficient) between all variables considered (in 49 infertile men).

	Sperm Concentration mL × 10^6^	Progressive Motility %	Normal Morphology %	Volume mL	Double-Stranded DNA%	Mature Chromatin %	F_2_-IsoPs ng/mL
Sperm Concentration mL × 10^6^	1						
Progressive Motility %	0.716 (*p* = 0.000)	1					
Normal Morphology %	0.745 (*p* = 0.000)	0.826 (*p* = 0.001)	1				
Volume mL	0.05 ns	0.022 ns	0.158 ns	1			
Double-Stranded DNA%	−0.063 ns	−0.093 ns	0.213 ns	0.059 ns	1		
Mature Chromatin %	0.356 ns	0.391 ns	0.461 (*p* = 0.001)	0.194 ns	−0.016 ns	1	
F_2_-IsoPs ng/mL	−0.156 ns	−0.151 ns	−0.084 ns	−0.284 Ns	0.570 (*p* = 0.000)	−0.643 (*p* = 0.000)	1

Sperm concentration: sperm/mL × 10^6^; progressive motility %: slow and rapid motility; normal morphology %: normal sperm morphology assessed by the Papanicolaou modified test, double-stranded DNA%: % of sperm green stained after acridine orange assay; mature chromatin %: % of unstained sperm after aniline blue test; and F_2_-IsoPs: F_2_ isoprostanes (ng/mL). Exact *p*-values have been reported in parentheses; ns represents that no significant *p*-values (after Bonferroni correction) were obtained.

**Table 2:** Logistic regression analysis of variables with respect to ART outcome. Legend: OR (odds ratio); CI (confidence interval).

Variables	Exp(B)/OR	Significance (*p*)	95% CI for Exp(B)/OR	
			Lower	Upper
Sperm Concentration × 10^6^	0.999	0.962	0.979	1.020
Volume mL	1.018	0.950	0.588	1.762
F_2_-IsoPs ng/mL	1.020	0.825	0.856	1.215
Mature Chromatin %	1.028	0.594	0.930	1.136
Normal Morphology %	0.710	0.058	0.498	1.012
Variable(s) entered in Step 1: Sperm Concentration mL × 10^6^; Volume (mL); F_2_-Isoprostanes (ng/mL); Mature Chromatin; Normal Morphology				
	Parameters tested for positive outcome			
Progressive Motility %	1.111	0.031	1.010	1.222
Double-Stranded DNA %	1.127	0.026	1.014	1.253

**Table 3:** Logistic regression analysis of variables with respect to embryo quality. Legend: OR (odds ratio); CI (confidence interval).

Variables	Exp(B)/OR	Significance (*p*)	95% CI for Exp(B)/OR	
			Lower	Upper
Sperm Concentration × 10^6^	0.994	0.473	0.977	1.011
Volume mL	1.386	0.208	0.834	2.303
Progressive Motility %	1.026	0.426	0.963	1.094
Mature Chromatin %	0.988	0.757	0.916	1.066
Normal Morphology %	0.904	0.341	0.735	1.113
Double-Stranded DNA %	1.029	0.397	0.964	1.098
Variable(s) entered in Step 1: Sperm Concentration mL × 10^6^; Volume (mL); Mature Chromatin; Normal Morphology; Progressive Motility; Double-Stranded DNA %				
	Parameters tested for embryo quality			
F_2_-IsoPs ng/mL	1.082	0.019	1.013	1.155

## Data Availability

The data are contained within this article and are available from the corresponding author upon request.
